# Phylogeny Reconstruction and Hybrid Analysis of *Populus* (Salicaceae) Based on Nucleotide Sequences of Multiple Single-Copy Nuclear Genes and Plastid Fragments

**DOI:** 10.1371/journal.pone.0103645

**Published:** 2014-08-12

**Authors:** Zhaoshan Wang, Shuhui Du, Selvadurai Dayanandan, Dongsheng Wang, Yanfei Zeng, Jianguo Zhang

**Affiliations:** 1 State Key Laboratory of Tree Genetics and Breeding, Key Laboratory of Silviculture of the State Forestry Administration, Research Institute of Forestry, Chinese Academy of Forestry, Beijing, People's Republic of China; 2 Forest and Evolutionary Genomics Laboratory, and the Centre for Structural and Functional Genomics, Biology Department, Concordia University, Montreal, Quebec, Canada; The New York Botanical Garden, United States of America

## Abstract

*Populus* (Salicaceae) is one of the most economically and ecologically important genera of forest trees. The complex reticulate evolution and lack of highly variable orthologous single-copy DNA markers have posed difficulties in resolving the phylogeny of this genus. Based on a large data set of nuclear and plastid DNA sequences, we reconstructed robust phylogeny of *Populus* using parsimony, maximum likelihood and Bayesian inference methods. The resulting phylogenetic trees showed better resolution at both inter- and intra-sectional level than previous studies. The results revealed that (1) the plastid-based phylogenetic tree resulted in two main clades, suggesting an early divergence of the maternal progenitors of *Populus*; (2) three advanced sections (*Populus*, *Aigeiros* and *Tacamahaca*) are of hybrid origin; (3) species of the section *Tacamahaca* could be divided into two major groups based on plastid and nuclear DNA data, suggesting a polyphyletic nature of the section; and (4) many species proved to be of hybrid origin based on the incongruence between plastid and nuclear DNA trees. Reticulate evolution may have played a significant role in the evolution history of *Populus* by facilitating rapid adaptive radiations into different environments.

## Introduction

The genus *Populus*, distributed throughout the northern hemisphere from subtropical to boreal forests [1] and one of the most economically and ecologically important genera of forest trees [2], is well known for its rapid growth, profuse vegetative propagation, environmental stress tolerance and the numerous uses of its wood [3]. Furthermore, the genus has become an excellent research model due to its small genome size and the completion of the genome sequence of *P. trichocarpa* [4]. A clear understanding of the evolutionary relationships of *Populus* species will provide an important foundation for biological studies and genetic breeding programs.

The relationships among sections as well as relationships within each section remain controversial and/or poorly resolved because of the extensive interspecific hybridization and high degree of morphological variation among species [3], [5]. The combination of these two features results in a major disagreement in the number of species and their delimitation [5], [6]. Eckenwalder [5] recognized 29 species of *Populus* grouped into six sections (*Abaso*, *Aigeiros*, *Leucoides*, *Populus*, *Tacamahaca*, *Turanga*) based on morphological similarity and crossability. However, more than 60 species (plus a number of hybrids, varieties and forms) are described in the Flora of China [6]. The morphology-based phylogenetic tree of *Populus* demonstrated that section *Abaso* and *Turanga* were sister groups to the other four sections followed by section *Leucoides*, but the relationship among the other three sections remain unresolved [5]. Phylogenetic analysis based on 5.8S RNA and ITS sequences suggested that section *Populus* was sister to *Leucoides*, *Tacamahaca* and *Aigeiros* [7]. On the other hand, a phylogenetic tree based on plastid RFLP data showed an opposite trend with section *Populus* as an advanced clade occupying the terminal position [8]. Cervera [9] proposed that *P. lasiocarpa* and *P. violascens* of section *Leucoides* should be classified into section *Tacamahaca*. Furthermore, the systematic placement of species in section *Aigeiros* is inconsistent [8], [10]. Cervera [9] proposed that *P. nigra* should be classified into a new section or as a subsection of section *Tacamahaca*. Most interspecific relationships within each section, in particular species in sections *Populus*, *Tacamahaca* and *Aigeiros*, are poorly resolved.

In addition to the lack of highly variable orthologous single-copy DNA markers, the complex reticulate evolution in *Populus* poses difficulty in resolving the phylogeny of *Populus* [8], [10], [11]. Species of *Populus* show extensive hybridization within sections as well as between closely-related sections. The species of section *Aigeiros*, *Tacamahaca* and *Leucoides* can intercross freely [12], [13]. The evidence for hybridization in *Populus* has been documented based on molecular markers, for example, in the phylogenetic study based on maternally inherited plastid and biparentally inherited nuclear DNA sequences, *P. nigra* showed different affinity to sections *Populus* and *Tacamahaca*, which suggested a possible hybrid origin for *P. nigra* [8], [10]. *P. tomentosa* has also been suspected to be a hybrid for a long time, but its exact parents remain unknown. The inconsistent systematic position of section *Populus* mentioned above may also be an indication of ancient hybridization in *Populus*.

The phylogenetic topology derived from maternally inherited plastid [14] data sets represents the maternal genealogy while nuclear DNA phylogeny mirrors biparental evolutionary history. Comparative analysis of DNA sequences from the nuclear and the plastid offers an effective way to parse out reticulate evolutionary events [8], [15]. The completion of the whole genome of *P. trichocarpa* [4] provides a means to find highly variable single-copy nuclear DNA sequences to assess the evolutionary relationships of *Populus*. Here, we utilized 24 single-copy nuclear DNA sequences and 12 plastid fragments to reconstruct the phylogeny of *Populus* with an emphasis on the reticulate evolution in the genus.

## Materials and Methods

### Ethics Statement

No special permits were required for this study and this study did not involve endangered or protected species.

### Species sampling, DNA extraction, PCR amplification and sequencing

The sampling was based on the classification proposed by Eckenwalder [5] and the Flora of China [6]. During the fieldwork from 2005 to 2013, we sampled twenty-six species representing 5 sections of *Populus* and 4 species of *Salix* as outgroups for the phylogeny reconstruction. The fresh leaves were dried and stored in silica gel. Information about the sampled species is given in (File S1 in Supporting Information S1).

Using a high throughput comparative genomic approach, Duarte et al. [16] identified 959 single-copy genes that were shared among *P. trichocarpa*, *Arabidopsis thaliana*, *Vitis vinifera* and *Oryza sativa* from which fifteen sequences were selected and characterized in our previous paper (Du et al., in press). The remaining nine pairs of primers were developed following the methods as described in Du et al. (in press). Twelve pairs of primers were selected from previous studies [17]–[21] and used for plastid amplification. All the primers used for amplifying and sequencing are listed in (File S2 in Supporting Information S1).

Total genomic DNA was isolated from silica-gel-dried leaves using a modified method [22]. Polymerase chain reaction (PCR) was performed in a total volume of 30 µL containing 5–50 ng of genomic DNA, 3 µL 10×PCR Buffer, 2.0 mM MgCl_2_, 0.8 µM of each dNTP, 2.4 µM of each primer and 0.15 U Ex Taq DNA polymerase (TaKaRa, Shiga, Japan). Amplification was carried out in a temperature gradient 96 U thermocycler (Applied Biosystems, Forster City, CA, USA), using following thermal cycling profiles: 4 min at 95°C followed by 30 cycles of 30 s at 94°C, 30 s at 52°C to 60°C (depending on the optimal annealing temperature of specific primers), 90 s at 72°C and a final extension at 72°C for 10 min. After purifying using a DNA Purification kit (Amersham Pharmacia Biotech, Piscataway, USA), the PCR product was sequenced using an ABI 3730 DNA analyzer (Applied Biosystems, Foster City, California, USA) with the same primers used for amplification. For the samples where direct sequencing failed, the purified PCR products were cloned into pGEM -T easy Vector System II (Promega, Madison, WI, USA). For each sample, 6–12 positive clones were randomly picked and sequenced in both directions using standard T7 and SP6 primers.

### Data analysis

The assembled contigs of each individual were aligned using CLUSTAL X [23] and refined manually in BioEdit [24]. For all the loci, regions with more than 5 mononucleotide or microsatellite repeats were excluded because of the uncertainty of homology which could be exacerbated by potential inaccuracies of enzymatic process during PCR and sequencing [25], [26]. All indels were excluded in the following phylogenetic analyses after coded as binary characters according to the simple indel coding method [27] using FastGap 1.2 [28].Phylogenetic relationships among species were reconstructed using parsimony, maximum likelihood (ML) and Bayesian methods. The parsimony analysis was conducted in PAUP* 4.0b10* [29], with all characters equally weighted and treated as unordered. Heuristic search was performed with MULPARS option, tree-bisection-reconnection (TBR) branch swapping, RANDOM stepwise addition with 1000 replicates and the number of trees held in RAM was set to be 100000. Bootstrap analysis was conducted to assess topological robustness with 1000 replicates using simple taxon addition [30]. An appropriate nucleotide substitution model for each sequence was determined using jModeltest [31]. The models were chosen according to the Akaike information criterion (AIC) and used for subsequent ML and Bayesian analysis. ML analysis was conducted in PAUP* 4.0b10* [29] with random taxon addition of 1000 replicates, TBR branch swapping, MULPARS option, 100000 trees held in RAM and 100 replication of bootstrap analysis. Bayesian inference was performed with MrBayes 3.2.1 [32]. Two independent runs of Metropolis-coupled MCMC were conducted simultaneously, with each run being one cold chain and three incrementally heated chains and all started randomly in the parameters space. All other parameters were set to default. 1,000,000 generations were run and trees were sampled once every 100 generations. The program Tracer v1.5 [33] was utilized to check for stationary. The first 25% of sampled trees were discarded as burn-in and the posterior probabilities were calculated from the remaining trees. All the phylogenetic trees were viewed in the program FigTree v 1.3.1 [34]. The homogeneity across nuclear DNA loci was tested using the Shimodaria-Hasegawa test [35] in CONSEL [36].

## Results

### Sequence Characteristics

We successfully obtained all 24 nuclear DNA sequences and 12 plastid fragments from all 30 species except for the locus DSH22 in *S. raddeana* and DSH11 in *P. grandidentata*, which failed to amplify and were treated as missing data in the subsequent phylogenetic analyses. After removing regions with mononucleotide repeats and microsatellite sequences, the aligned length of the nuclear DNA ranged from 222 bp to 1106 bp with a total length of 15732 bp, in which exon sequences consisted of 10184 bp (64.7%). As shown in (File S3 in Supporting Information S1), the number of variable sites ranged from 38 (locus DSH6) to 160 (locus DSH10) and that of informative sites ranged from 27 (locus DSH6) to 111 (DSH22). The aligned length of plastid fragments varied between 532 bp and 2620 bp with a total length of 14197 bp, the exon sequences of which only occupied 4592 bp (32.3%). The most appropriate models fitted each locus decided by jModeltest [31] was presented in (File S3 in Supporting Information S1).

### Phylogenetic analysis of the plastid fragments

**Figure 1:**
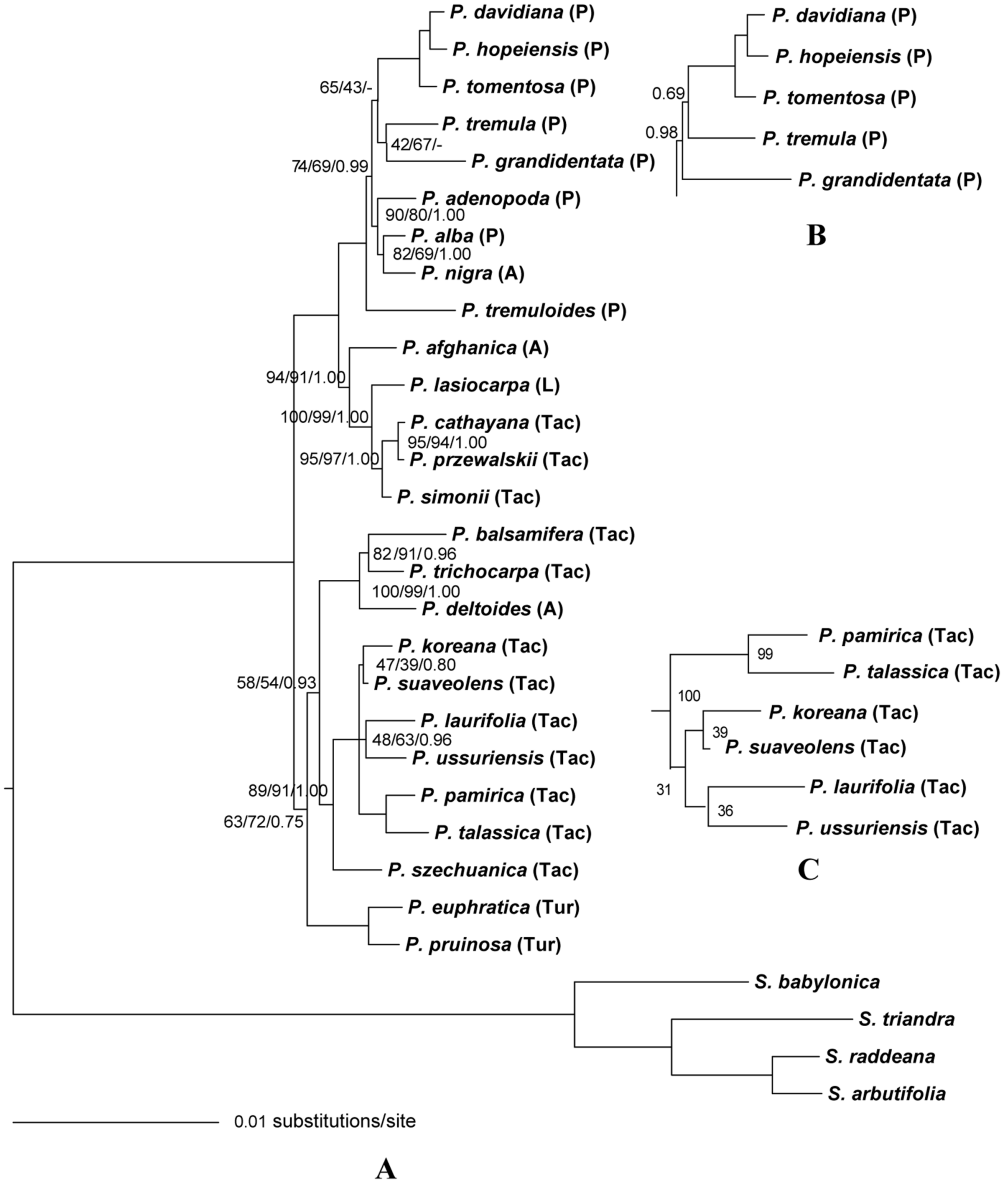
Phylogeny of *Populus* obtained from the combined 12 plastid fragments using parsimony method, (A). Numbers next to nodes sequentially indicated ML/parsimony/BI support values. The branches without numbers indicate 100% statistical support. (B) The topology difference derived from Bayesian analysis while (C) is from ML analysis. A, *Aigeiros*; L, *Leucoides*; P, *Populus*; Tac, *Tacamahaca*; Tur, *Turanga*.

Phylogenetic relationships among species remained poorly resolved based on individual plastid sequences (File S5 in Supporting Information S1). Because all plastid gene sequences are effectively inherited as one locus, they were concatenated into a single contiguous sequence for the phylogenetic analysis. The best fitting evolutionary model for the combined plastid data set was TVM+G in ML and Bayesian analyses. 50% majority-rule consensus parsimony and ML trees were generated from 8 most parsimonious trees and 6 most likely trees based on the combined plastid data set. The phylogenetic trees generated by parsimony, ML and Bayesian methods were similar to each other, with only a few differences in bootstrap support (BS) or posterior probability (PP) values in some clades. For instance, the sister relationship between *P. tremula* and *P. grandidentata*, and between *P. tomentosa* and *P. tremula/P. grandidentata* in the parsimony and ML trees were not supported by Bayesian methods (Figure 1).

As shown in Figure 1, all *Populus* species formed a fully supported monophyletic group comprising two major clades. In the first clade, all species of section *Populus* and *P. nigra* of section *Aigeiros* formed a single group with *P. tremuloides* sister to other species. Within this group, *P. davidiana*, *P. hopeiensis* and *P. tomentosa* grouped together sister to *P. tremula* and *P. grandidentata*, meanwhile, *P. alba*, *P. nigra* and *P. adenopoda* showed close phylogenetic relationships to each other. Unexpectedly, *P. cathayana*, *P. simonii* and *P. przewalskii* of section *Tacamahaca*, *P. lasiocarpa* of section *Leucoides* and *P. afghanica* of section *Aigeiros* formed the other highly supported group. In the second clade, *P. pruinosa* and *P. euphratica* of section *Turanga* clustered together as sister taxa to other species. Three American poplars, *P. balsamifera*, *P. trichocarpa* and *P. deltoides* were more closely related to each other than to other Asiatic *Tacamahaca* species which further divided into two subclades with *P. szechuanica* sister to a group of species comprising *P. koreana*, *P. suaveolens*, *P. laurifolia*, *P. ussuriensis*, *P. pamirica* and *P. talassica*.

### Phylogenetic analysis of the nuclear DNA

Phylogenetic relationships based on individual nuclear DNA loci were not fully resolved (File S6 in Supporting Information S1). The Shimodaria-Hasegawa test showed that there was no significant incongruence among most of the individual nuclear DNA loci (P>0.05), but slight incongruence was detected in some cases (File S4 in Supporting Information S1). After excluding these incongruent loci from the phylogeny reconstruction, topology of the phylogenetic tree remained the same with only some differences in BS or PP value. We, therefore, combined the 24 individual nuclear DNA sequences to a single data set to reconstruct the phylogeny of *Populus* and to make direct comparison with the cpDNA phylogeny.

**Figure 2:**
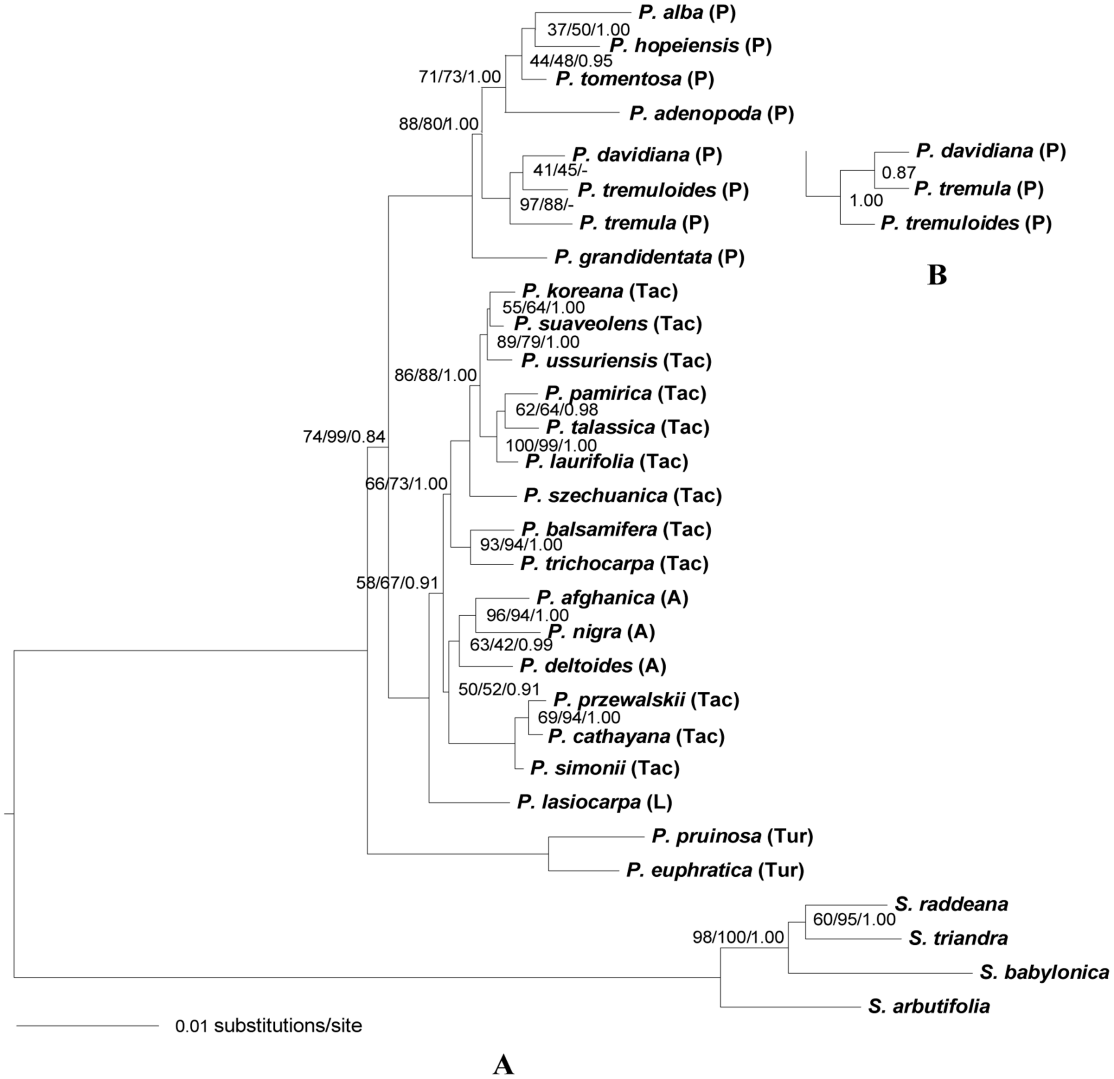
Phylogeny of Populus obtained from the combined 24 single-copy nuclear DNA sequences using ML method, (A). Numbers next to nodes sequentially indicate ML/parsimony/BI support values. The branches without numbers indicate 100% statistical support. (B) The topology difference derived from Bayesian analysis. A, *Aigeiros*; L, *Leucoides*; P, *Populus*; Tac, *Tacamahaca*; Tur, *Turanga*.

The best fitting evolutionary model for the combined nuclear DNA data set was GTR+R in ML and Bayesian analyses. The topologies of parsimony and ML trees are from 50% majority-rule consensus trees, which were generated from 4 most parsimonious trees and 6 most likely trees, respectively, based on combined nuclear DNA data set. Nuclear DNA phylogenetic trees generated from parsimony, ML and Bayesian methods showed similar topologies to each other with the only differences within section *Populus* of Bayesian tree (Figure 2). In the phylogenetic tree generated using nuclear data set (Figure 2), monophyly of *Populus* was strongly supported. Section *Turanga* was sister to other sections, followed by section *Populus* with high support value, in which a native North America poplar, *P. grandidentata* was sister to other species. The remaining species of section *Populus* subdivided into two groups: *P. tomentosa*, *P. alba*, *P. hopeiensis* and *P. adenopoda* clustered together with slightly lower resolution in the first group and three tremble aspens, *P. davidiana*, *P. tremuloides* and *P. tremula* showed close relationship to each other with high statistical support in the second. Section *Leucoides* (*P. lasiocarpa*) followed section *Populus* and was sister to sections *Tacamahaca* and *Aigeiros*. All the species of these two sections subdivided into two subclades. In the first subclade, *P. cathayana*, *P. simonii* and *P. przewalskii* clustered together sister to section *Aigeiros*, within which all the three species clustered as a single clade. The second subclade consisted of two sister groups: the two North America species (*P. balsamifera/P. trichocarpa*) and the other 7 species.

## Discussion

### Phylogenetic relationships of *Populus*

With a large combined data set of 24 nuclear DNA sequences and 12 plastid fragments, we reconstructed maternal and biparental phylogenies of *Populus* (Figure 1 and 2). All species of *Populus* clustered together as one clade separated from outgroup species, which supported the results of previous studies [5], [10], [37], [38]. Species of each section grouped together in both nuclear and plastid DNA phylogenies with certain exceptions. Species of section *Aigeiros* clustered within different clades in plastid tree. *P. simonii*, *P. cathayana* and *P. przewalskii* of section *Tacamahac*a grouped with *P. lasiocarpa* separating from other balsam poplars in the plastid phylogeny.

There has been an argument about the oldest lineage of *Populus* for a long time. Phylogenetic analysis based on AFLP and ITS sequence proposed that section *Populus* was the oldest lineage in *Populus* [9]. However, fossil records and morphological phylogenetic analysis showed that section *Turanga* was sister to other sections [5]. *Populus wilmottae*, one of the most ancient fossil species of *Populus* [39], bears three-valved capsules, a feature considered primitive based on its predominance in the Violales, including many Flacourtiaceae, which is shared by section *Turanga* [5], [39]. Furthermore, the modern *Turanga* lineage is strikingly heteroblastic, with willow-like juvenile leaves strongly differentiating it from other species of *Populus* — thought to have developed early in the evolutionary history of this genus [5]. This suggested that section *Turanga* is an ancestral lineage as observed in phylogenetic trees with high BS or PP support value.

Intersectional relationships of *Populus* have been poorly resolved because of inadequate samples and/or insufficient resolution of molecular markers used in previous studies. Eckenwalder [5] reconstructed the phylogeny of *Populus* using 76 morphological characters. Section *Abaso* and *Turanga* were sister to other sections followed by section *Leucoides* and the relationship among the three advanced sections remained unresolved [5]. In an ITS sequence-based phylogeny of *Populus*, section *Populus* was monophyletic and species of sections *Tacamahaca* and *Aigeiros* mixed together in one clade with low statistical support [40].The relationship between the two sections did not resolve in *trnT-trnF* phylogeny [10]. In the phylogenetic tree reconstructed based on AFLP data, Cervera et al. [9] found that section *Leucoides* showed close relationships with sections *Tacamahaca* and *Aigeiros*. Moreover he proposed that *P. ciliata*, *P. lasiocarpa* and *P. violascens* of section *Leucoides* should be classified into section *Tacamahaca* [9]. ISSR-based phylogeny of *Populus* also revealed close intersectional relationship between section *Tacamahaca* and *Aigeiros* [37].

Based on the nuclear DNA phylogeny, section *Populus* and *Leucoides* were successive to *Turanga*, section *Tacamahaca* and *Aigeiros* occupied the terminal position. This phylogenetic order more or less followed previously published patterns [5]. The close affinity between section *Tacamahaca* and *Aigeiros* observed in the present study is in agreement with previous studies [5], [10], [32], [34]. For the first time, we found that species of *Populus* divided into two major clades in the plastid phylogeny, which reflected an early divergence of the maternal progenitors of *Populus*.

### Intrasectional phylogenetic relationships

In previous studies, interspecific relationships within sections of *Populus* were rarely addressed or poorly resolved [9], [10]. Based on our data, intrasectional relationships among species were relatively better resolved. Because only one species of section *Leucoides* and two species of section *Turanga* were utilized in analysis, interspecific relationships of these two sections cannot be assessed. Furthermore, interspecific relationships of section *Aigeiros* is discussed below with an emphasis on hybrid origin.

#### Section Populus

The results from both nuclear and plastid DNA analyses suggest that section Populus is monophyletic. In the nuclear DNA tree, P. grandidentata was sister to other species which subdivided into two clades. The North American trembling aspen P. tremuloides showed close genetic affinity to Eurasian aspen P. tremula and P. davidiana with high BS or PP value. These three species are similar to each other with respect to morphological characters [6], [41]. Eckenwalder [5] even proposed that P. tremuloides, P. tremula and P. davidiana should be merged into a single species. The rationalization of this hypothesis and the accurate time of origin and differentiation among the three species require further analysis with a larger sample size. The remaining four species group in a single clade in keeping with their morphological similarity. Unlike the nuclear DNA tree, another North American aspen, P. tremuloides, separated from other species and formed a single clade in the plastid tree, which indicated that P. tremuloides was of an ancestral maternal origin. As in the nuclear DNA phylogeny, P. adenopoda and P. alba show closest affinity to each other whereas they are widely distributed in southern China and central Eurasia, respectively. It is of great interest to pay attention to allopatric speciation resulted from geographic isolation between these two species in future.

#### Section Tacamahaca

The relationships of species within section Tacamahaca are known to be the most complicated. In present study, P. cathayana, P. przewalskii and P. simonii clustered with section Leucoides in the plastid-based tree but sister to section Aigeiros in nuclear DNA tree as a single highly supported group. The classification of P. cathayana, P. przewalskii and P. simonii either in section Tacamahaca or in other sections requires further investigation based on comprehensive sample with morphological and molecular methods. The other balsam poplars formed two sister clades. Two North American balsam poplars P. trichocarpa and P. balsamifera formed a strongly supported clade sister to the Asian ones in the plastid and nuclear DNA phylogeny considering their morphological, ecogeographic similarity and recent divergence [42]. A similar situation was shown in Asiatic species of section Tacamahaca; however, their distribution is allopatric in China. We speculate that these species derived from vicariance and allopatric divergence from a once widely-distributed ancestral species of section Tacamahaca. The polyphyly of section Tacamahaca was clearly verified based on our plastid and nuclear DNA phylogeny [10], which was in agreement with morphology-based phylogenetic analysis [5].

### Reticulate evolution in *Populus*

The most ancient undisputed fossil record of *Populus* dated to late Paleocene (about 58 Ma) is considered to be related to section *Turanga* because of the morphological similarity [39], [43]. Through combined fossil record and phylogenetic analyses we ascertain that section *Turanga* is more primitive in *Populus*. Species of the section *Leucoides* which inhabit permanent swamps first appeared in North America in late Eocene [5]. Combining fossil records and the phylogenetic tree based on 76 morphological characters, Eckenwalder [5] speculated that the temperate habitats of *Populus* were invaded by an ancestral member of section *Leucoides*, and following this, there was a rapid radiation (effectively simultaneous) driven by ancient hybridization events into the distinct habitats along with appearance of other advanced sections. The newly appeared sections can adapt to more extreme environments, for example, section *Populus* can tolerate aridity and coldness. The subsequent evolution within each section were partly influenced by hybridization [1].

#### Section Populus

Based on our plastid and nuclear DNA phylogeny, the ancestor of section Populus originated from the hybridization of two ancestral sections (section Turanga and Leucoides) with section Leucoides as the maternal parent. In the plastid tree, P. tremuloides was sister to other species in section Populus. In the nuclear DNA tree, P. tremuloides clustered with the Asiatic trembling aspen. According to leaf morphological variation and the fossil record, P. tremuloides was hypothesized to be a species of complex origin derived from multiple ancestral hybridization [5], [44]. It is inferred that hybridization occurred between ancestors of P. davidiana and P. tremula dispersed from Eurasia to North America as the paternal parent and P. tremuloides.

*P. tomentosa*, one of the cultivated species widely distributed in China, has attracted attention from taxonomists and geneticists [45]. Based on morphological traits and molecular evidence, *P. tomentosa* is considered as a complex hybrid species involving more than two species. Both RAPD and AFLP analysis suggests that *P. tomentosa* has closest affinity to *P. adenopoda* and was possibly a natural hybrid of *P. alba* and *P. adenopoda* [46]. The nuclear DNA phylogeny also revealed the close relationship between *P. tomentosa* and *P. adenopoda*. However, *P. adenopoda* and *P. tomentosa* clustered in two different clades within section *Populus* in the plastid phylogeny. In the hybridization event giving rise to *P. tomentosa*, the ancestor of *P. davidiana* and *P. hopeiensis* served as the maternal parent and *P. adenopoda* as the paternal role. Based on morphological similarity to *P. hopeiensis* and the closer genetic affinity, we infer that *P. tomentosa* may have been domesticated from *P. hopeiensis*, which shows sympatric distribution in China.

#### Section Tacamahaca

Twelve species of section Tacamahaca were used in this study. P. cathayana, P. przewalskii and P. simonii clustered with section Leucoides based on plastid phylogeny and sister to section Aigeiros in nuclear DNA tree, which indicated that their maternal lineage derived from section Leucoides followed by high gene flow with section Aigeiros. The remained nine species of section Tacamahaca showed a close affinity to section Turanga in the plastid tree and sister to section Leucoides in nuclear DNA tree, which suggested these species also derived from a hybridization event in which section Turanga and section Leucoides played the maternal and paternal roles, respectively.

#### Section Aigeiros

Three species of section Aigeiros were used in this study. They clustered together in the nuclear DNA tree, however, in the plastid tree P. nigra clustered with P. alba, P. deltoids grouped with P. balsamifera and P. trichocarpa while P. afghanica showed close affinity to P. lasiocarpa. This implied that all of the three species of section Aigeiros were hybrid origin with different maternal parent.

Despite large number of morphological, molecular and phylogenetic studies on the hybrid origin of *P. nigra*, the maternal parent of this species remains uncertain. Phylogenetic analysis based on plastid data clustered *P. nigra* within section *Populus* and the plastid of *P. nigra* show close affinity to either *P. alba* [8] or common ancestors of *P. tremula* and *P. davidiana* [10]. Our results confirm that the plastid of *P. nigra* is inherited from *P. alba* or a common ancestor shared with *P. alba*. A close relationship between *P. deltoides* and section *Tacamahaca* was observed previously [9], [10], [37], [47]. In the plastid tree, *P. deltoides* along with two native North American balsam poplars of section *Tacamahaca* form a monophyletic group with high BS or PP values. The similarity of floral morphology [48], overlapping distribution [49] and hybridization [50]–[52] of these three species in North America suggest that they may have diverged from a common maternal ancestor following a hybridization event giving rise to *P. deltoides*. A similar situation was also seen in *P. afghanica*, which shares a common maternal ancestor with *P. lasiocarpa*.

The taxonomic position of species in section *Aigeiros* is contentious. Rajora & Dancik [47] proposed a new section *Nigrae*, consisting only of *P. nigra*. Cervera *et al.* [9] pointed out that *P. deltoides* should be separated from the consectional *P. nigra*. Moreover, Eckenwalder [53] suggested that sections *Tacamahaca* and *Aigeiros* should be merged into a single section because of their close evolutionary relationships, which was supported by phylogenetic analysis based on ITS and plastid *trnT-trnF* sequences of *Populus* [10]. Nevertheless, considering the highly supported relationship in our nuclear DNA phylogeny and morphological similarity among the species of section *Aigeiros*, it is reasonable to retain this section which is a good model for further research about hybrid speciation in *Populus*.

In all, species of section *Populus* and *Tacamahaca* played a part in the origin of species in section *Aigeiros*, which suggests that section *Aigeiros* may have originated later than the former two sections. This is consistent with the paleontological results, and fossils of the section *Aigeiros* occur later in the sediments [5].

## Conclusions

Based on nucleotide sequences of 24 single-copy nuclear genes and 12 plastid fragments, two robust phylogenies of *Populus* (Salicaceae) were reconstructed. The genus *Populus* was monophyletic in both phylogenetic trees and section *Turanga* was an ancestral lineage within the genus *Populus.* Comparative analyses of these two phylogenetic trees revealed reticulate evolutionary patterns in this genus. Three advanced sections (*Populus*, *Aigeiros* and *Tacamahaca*) were of hybrid origin. A detailed study involving more species (especially section *Abaso*) and genes are needed to further infer the origin, dispersal and hybridization in *Populus*.

## Supporting Information

Supporting Information S1 **Combined supporting information file.** File S1. The information of species (names, altitude, longitude, latitude, collector, collection number and place of voucher deposition). File S2. The information of primers. File S3. The information of nuclear and plastid DNA sequences. File S4. Results of the Shimodaira-Hasegawa test that are significant incongruence (P<0.05). File S5. Phylogenetic trees reconstructed based on plastid fragments using parsimony, most likelihood and Bayesian inference methods. Numbers next to nodes indicated bootstrap support value or posterior probabilities. File S6. Phylogenetic trees reconstructed based on nuclear DNA fragments using parsimony, most likelihood and Bayesian inference methods. Numbers next to nodes indicated bootstrap support value or posterior probabilities.
(RAR)
